# From Ethnobotany to Biotechnology: Wound Healing and Anti-Inflammatory Properties of *Sedum telephium* L. In Vitro Cultures

**DOI:** 10.3390/molecules29112472

**Published:** 2024-05-24

**Authors:** Vanessa Dalla Costa, Anna Piovan, Raffaella Filippini, Paola Brun

**Affiliations:** 1 Department of Pharmaceutical and Pharmacological Sciences, University of Padova, Via Marzolo 5, 35131 Padova, Italy; vanessa.dallacosta@phd.unipd.it (V.D.C.); anna.piovan@unipd.it (A.P.); raffaella.filippini@unipd.it (R.F.); 2 Department of Molecular Medicine, University of Padova, Via Gabelli 63, 35121 Padova, Italy

**Keywords:** *Sedum telephium*, plant cell cultures, kaempferol, quercetin, anti-inflammatory, wound-healing, biotechnology

## Abstract

*Sedum telephium* is a succulent plant used in traditional medicine, particularly in Italy, for its efficacy in treating localized inflammation such as burns, warts, and wounds. Fresh leaves or freshly obtained derivatives are directly applied to the injuries for these purposes. However, challenges such as the lack of microbiologically controlled materials and product standardization prompted the exploration of more controlled biotechnological alternatives, utilizing in vitro plant cell cultures of *S. telephium*. In the present study, we used HPLC-DAD analysis to reveal a characteristic flavonol profile in juices from in vivo leaves and in vitro materials mainly characterized by several kaempferol and quercetin derivatives. The leaf juice exhibited the highest content in total flavonol and kaempferol derivatives, whereas juice from callus grown in medium with hormones and callus suspensions showed elevated levels of quercetin derivatives. The in vitro anti-inflammatory and wound-healing assays evidenced the great potential of callus and suspension cultures in dampening inflammation and fostering wound closure, suggesting quercetin may have a pivotal role in biological activities.

## 1. Introduction

*Hylotelephium telephium* (L.) H. Ohba is a succulent perennial plant belonging to the Crassulaceae family, probably best known by its basionym *Sedum telephium* L. It is native to Eastern Europe, China, and Japan but has been introduced into many other parts of the world [1]. *S*. *telephium* is also widespread in the Italian flora as a spontaneous and cultivated species, mainly for its ornamental value. *S. telephium* has been widely known since ancient times for its anti-inflammatory activity when topically applied [2], but it has never received significant interest among the scientific community. Indeed, the scientific literature is scarce. Nonetheless, *S*. *telephium* is well known in Italian ethnobotany, primarily for treating burns, ulcers, warts, abscesses, and wounds [3]. The first evidence of anti-inflammatory, keratolytic, and analgesic activities of leaves from *S. telephium* L. ssp. *maximum* Schinz & Thell. has been confirmed by experiments carried out at the Emergency Unit of the Torre Galli Hospital (Florence, Italy). The leaves without the external cuticle or homogenate fresh leaves were usually applied topically to painful wounds, burns, and eczemas to promote healing and reduce inflammation and pain [4]. Subsequently, studies clarified the constituents of *S*. *telephium* and the anti-inflammatory and antioxidant activities.

The anti-inflammatory activity of the crude extracts or fractions from *S. telephium*, tested on in vitro and in vivo models, seems to be mainly related to the polysaccharides present at the leaf level [5]. Lately, other authors [6] have demonstrated the anti-inflammatory activity of a methanolic extract of *S*. *telephium* containing both the polysaccharide and the flavonolic fractions, suggesting the contribution of the flavonol glycosides to the anti-inflammatory activity. The wound-healing effects of extracts from *S. telephium* are still debated. Total juice from fresh leaves has been reported to inhibit cell adhesion to the extracellular matrix. Further analysis revealed that while flavonols were inactive, the polysaccharide fractions were endowed with anti-adhesive effects [7]. In vitro antioxidant and in vivo skin photoprotective effects of three lyophilized extracts obtained from fresh leaf juice were evaluated, revealing that both the total lyophilized juice and, mainly, the lyophilized flavonolic fraction significantly protected against oxidative damage. In contrast, the polysaccharide fraction was ineffective [8]. Even though the scientific community has recently focused its attention on the phytochemical composition of *S*. *telephium,* aiming at elucidating the fractions involved in the biological activities, the literature has been focused on the ethnobotanical way of preparation, namely, using fresh leaves as starting material to obtain fresh juices or extracts or applying them “as they are” on the injuries to promote wound healing.

Using fresh material is a simple and “natural” approach but poses problems, such as the year-round supply, non-sterility, and poorly controlled plant material. In the latter case, the topical application on damaged skin may increase the risk of secondary infections. In this contest, plant cell cultures are an attractive alternative for producing valuable materials with promising applications in the healthcare and cosmetic sectors [9,10,11,12] as they present some relevant advantages. Plant cell cultures are independent of geographical, seasonal, and environmental variations, and they offer a defined production system, which ensures a continuous supply of products, uniform quality, and yield [13,14]. Moreover, cell cultures are free from contamination by other organisms, pesticides, and insecticides [15,16]. Therefore, the possibility of using in vitro cultures of *S. telephium* represents an intriguing alternative to fresh materials, ensuring the same biological activities but overcoming the weaknesses of using leaves from in vivo plants. In this study, we evaluated the wound healing and anti-inflammatory properties of juice obtained from in vivo leaves and juices obtained from in vitro plantlets and undifferentiated cells, calli, and suspensions for a possible application in the medical field.

## 2. Results

### 2.1. HPLC Fingerprint Analysis

**Figure 1:**
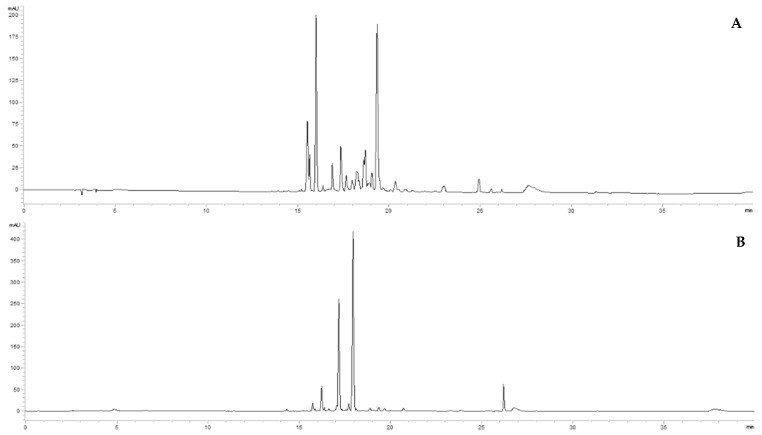
Chromatogram of the leaves’ juice (**A**) and chromatogram of the callus’s juice (**B**) acquired at 365 nm.

Preliminary HPLC-DAD analyses were performed to obtain the chromatographic fingerprints and define marker compounds for the quantitative analysis. Juice from in vivo leaves and juices from in vitro materials (plantlets, calli, and suspensions) showed a chromatographic profile at 365 nm characterized by a cluster of peaks between 15 and 28 min. Figure 1 reports a chromatogram of the leaves’ juice as an example of in vivo material (Figure 1A) and a chromatogram of the callus’s juice, grown in S10 medium, for in vitro material (Figure 1B). The chromatographic analyses of the samples highlighted a different metabolic profile.

**Figure 2:**
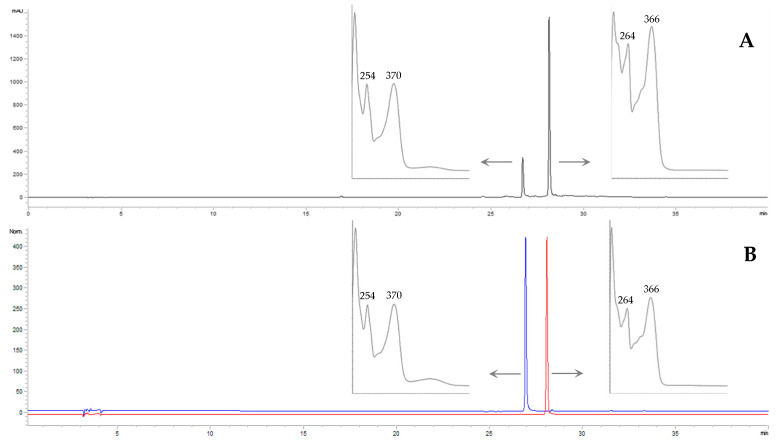
Chromatogram of leaves’ juice acid hydrolysis (**A**) compared with chromatograms of quercetin (blue) and kaempferol (red) standards (**B**), acquired at 365 nm. Spectra of quercetin (λ_max_ 254, 370 nm) are reported for peaks at RT 26.7 min, and spectra of kaempferol (λ_max_ 264, 366 nm) are reported for peaks at RT 28.1 min.

Considering the UV spectra of the peaks in this chromatographic frame, they exhibited an absorption peak in the range 300–380 (band I) associated with the absorption due to the B-ring cinnamoyl system and an absorption peak in the range 240–280 (band II), associated with the A-ring benzoyl system [17]. These absorptions being peculiar to flavone and flavonol structures and based on literature data in which only kaempferol and quercetin derivatives are reported in *S*. *telephium*, the acid hydrolysis of the samples was performed in order to release the free aglycons. The analysis of the hydrolyzed extracts confirmed quercetin and kaempferol as the only aglycones, as previously reported in the literature [2,18]. In Figure 2, we present a representative chromatogram of the leaves’ juice acid hydrolysis compared with the chromatogram of kaempferol and quercetin standards, which reported retention times (RTs) of 26.7 and 28.1 min, respectively.

Six juices were analyzed: juice from in vivo leaves (LE-J), in vitro plantlets (PL-J), calli cultured in medium S10 (C1-J), calli cultured in SH (C2-J), cell suspension in S10 (S1-J), and cell suspension in SH (S2-J). Analyzing the absorption spectra of the peaks within the RT from 15 to 28 min of the non-hydrolyzed juices, comparing them with kaempferol and quercetin spectra and based on published data, it was settled that all the cluster peaks were kaempferol derivatives and, to a lesser extent, quercetin derivatives, as confirmed by the analysis of hydrolyzed extracts in which kaempferol was predominant (Figure 2A). The kaempferol and quercetin derivatives observed in the six analyzed juices, with the respective RT, are reported in Table 1.

The qualitative fingerprint highlights that some compounds are present in all juices, whereas others are restricted to juices obtained from differentiated in vivo and in vitro material and others to calli and/or suspensions.

### 2.2. Quantitative Analysis

**Figure 3:**
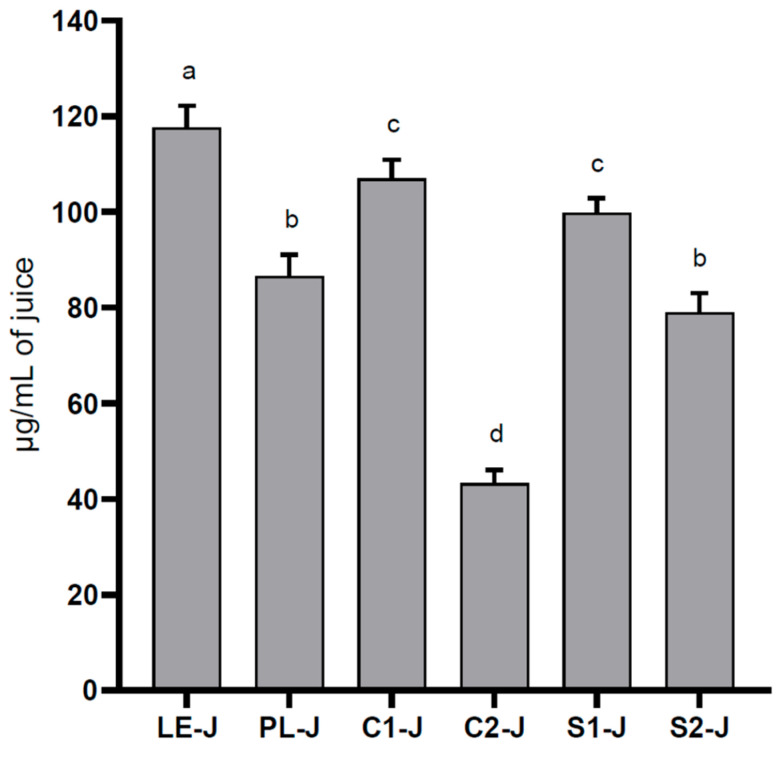
Total flavonol content in LE-J, PL-J, C1-J, C2-J, S1-J, and S2-J, calculated as kaempferol and quercetin equivalents. Results are expressed as mean ± SD, *n* = 3. The significant differences at *p* < 0.05 are denoted by different Latin letters.

Based on the qualitative analyses, the total flavonol content was calculated by summing up the concentration of kaempferol derivatives and quercetin derivatives. In Figure 3, we provide the total flavonoid contents of the juices, calculated as kaempferol and quercetin equivalents. LE-J had the highest content (117.7 ± 4.5 µg/mL), significantly different from all the other samples. C1-J was not statistically different from S1-J (*p* > 0.05), whereas C2-J was statistically different from S2-J, and the latter was comparable with PL-J. The lowest content of flavonoid was found in C2-J, which reported a significantly lower content (43.3 ± 2.8 µg/mL) than all samples (*p* < 0.001).

**Figure 4:**
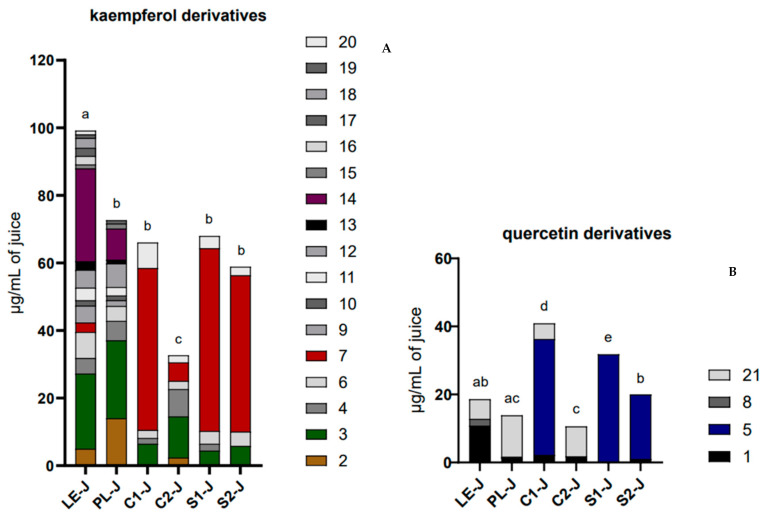
Kaempferol (**A**) and quercetin (**B**) derivative content in LE-J, PL-J, C1-J, C2-J, S1-J, and S2-J. Results are expressed as mean, *n* = 3. The significant differences at *p* < 0.05 are denoted by different Latin letters.

To reveal the contributions of kaempferol and quercetin derivatives to the total flavonol content, we report their respective contents in Figure 4A,B. In each histogram, it is also possible to appreciate the single compounds contributing to the total quantity.

LE-J had the highest content of kaempferol derivatives of all the samples (*p* < 0.001); on the other hand, LE-J showed a relatively low content of quercetin derivatives, non-statistically different from PL-J and S2-J. PL-J showed a kaempferol derivative content lower than LE-J but equal to C1-J, S1-J, and S2-J and a quercetin derivative content non-statistically different from both LE-J and C2-J. C1-J and S1-J resulted to be the two juices with the highest content of quercetin derivatives, 40.9 ± 4.0 µg/mL for C1-J and 31.8 ± 1.5 µg/mL for S1-J. As mentioned, S2-J shows a comparable content of kaempferol derivatives with respect to S1-J, C1-J, and C2-J but not for quercetin derivatives. C2-J was the poorest juice for both kaempferol and quercetin derivatives, different from all the other samples for the kaempferol derivatives (*p* < 0.01/0.001), and similar to PL-J for quercetin derivative content.

The qualitative analysis highlighted differences among the samples, especially about the juices derived from differentiated and undifferentiated material. Looking at the quantitative profile of kaempferol derivatives, we observed that LE-J, the most qualitatively rich juice, was characterized by **3** and **14** as the prevalent compounds. In PL-J, compound **3** was the predominant compound, followed by compounds **2** and **14**. C1-J, S1-J, and S2-J showed a smaller number of kaempferol derivatives when compared with LE-J and PL-J and very similar profiles, in which compound **7** sharply prevailed, detected in LE-J only in small amounts. Compound **3** was present in a three to four times lower quantity than in the LE-J and PL-J samples. C2-J, although very similar from a qualitative point of view to C1-J, S1-J, and S2-J, showed a content of compound **7** that was about eight to nine times lower when compared with the same samples.

On the other hand, by observing the quantitative profile of quercetin derivatives, much less complex in all the samples compared with kaempferol derivatives, it can be noted that in LE-J, compound **1** was the dominant one, while compound **21** prevailed in PL-J and C2-J. This differed from the quali-quantitative profile of C1-J, S1-J, and S2-J, where compound **5** prevailed but was never detected in the other samples.

### 2.3. Identification of Non-Toxic Concentrations of S. telephium Juices

**Figure 5:**
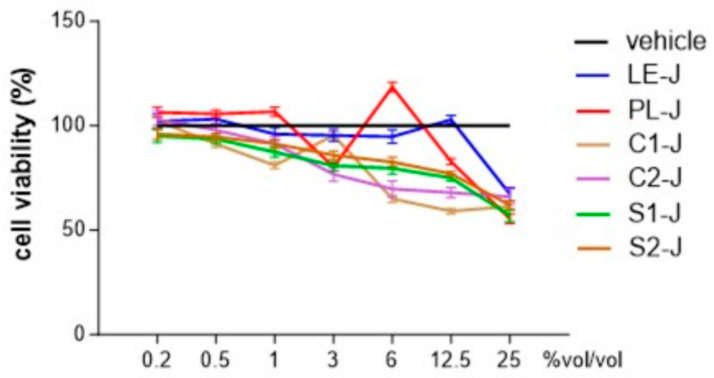
MTT assay of *S. telephium* juices at different concentrations. Data are reported as mean ± SE of three independent experiments, each performed in triplicate.

We investigated the cytotoxic activities of *S. telephium* juices in human primary differentiated macrophages using the MTT assay. Extracts were tested at concentrations ranging from 0.2 to 25% *vol*/*vol*, and the cell viability was calculated over the values obtained from cells treated with the vehicle alone. As reported in Figure 5, all the tested extracts reported a percentage of cell viability comparable to the vehicle-treated cells when tested at concentrations equal to or less than 1% *vol/vol*. When tested at concentrations higher than 3% *vol/vol,* almost all the extracts significantly reduced the cell viability. As the 1% *vol/vol* was the highest non-toxic concentration for all the extracts, we used the extracts at this concentration in the subsequent experiments.

### 2.4. C1-J, S1-J, and S2-J Induce Wound Repair

**Figure 6:**
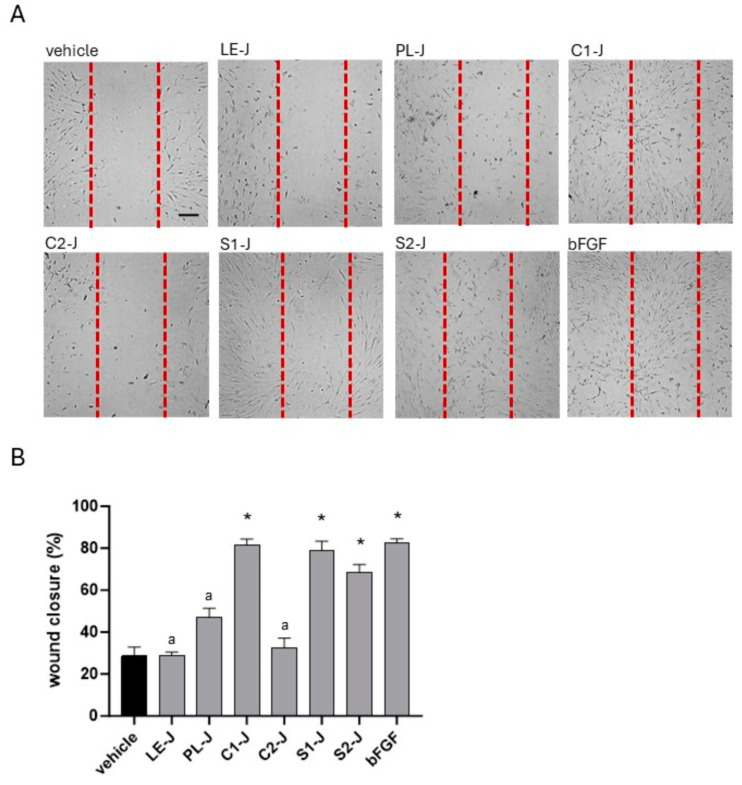
Panel (**A**). Wound healing in HFF1 cells treated for 24 h with 1% *vol*/*vol S. telephium* juices or bFGF (10 ng/mL). Representative images are reported. Images were acquired using an optical microscope equipped with a camera. Scale bar = 100 µm. Panel (**B**). The distance between cells at the edges of the scratch was measured using the software ImageJ (version 1.54h) and expressed as the percentage of closure of the area compared with cells incubated with the vehicle. Data are reported as mean ± SE of two independent experiments, each performed in triplicate. * denotes *p* < 0.05 vs. vehicle-treated cells; ^a^ denotes *p* < 0.05 vs. C1-J-treated cells, S1-J-treated cells, S2-J-treated cells, and bFGF-treated cells.

Using the scratch assay, we investigated the ability of *S. telephium* juices to stimulate wound closure in HFF1 cells. As reported in Figure 6, cells cultured with C1-J, S1-J, and S2-J migrated in the artificial wound areas compared with vehicle-treated cells. The extent of cell migration was comparable to cells incubated with bFGF. Slight effects in migration were reported in cells cultured with PL-J, whereas we did not observe evident cell migration in samples incubated with LE-J and C2-J. Indeed, the percentage of wound closure was 81.66% for C1-J, 79.1% in S1-J-treated cells, and 68.66% in S2-J-treated cells. In vehicle-treated cells, the percentage of wound closure was 28.66%.

### 2.5. C1-J, S1-J, and S2-J Report Anti-Inflammatory Activity in Human Macrophages

**Figure 7:**
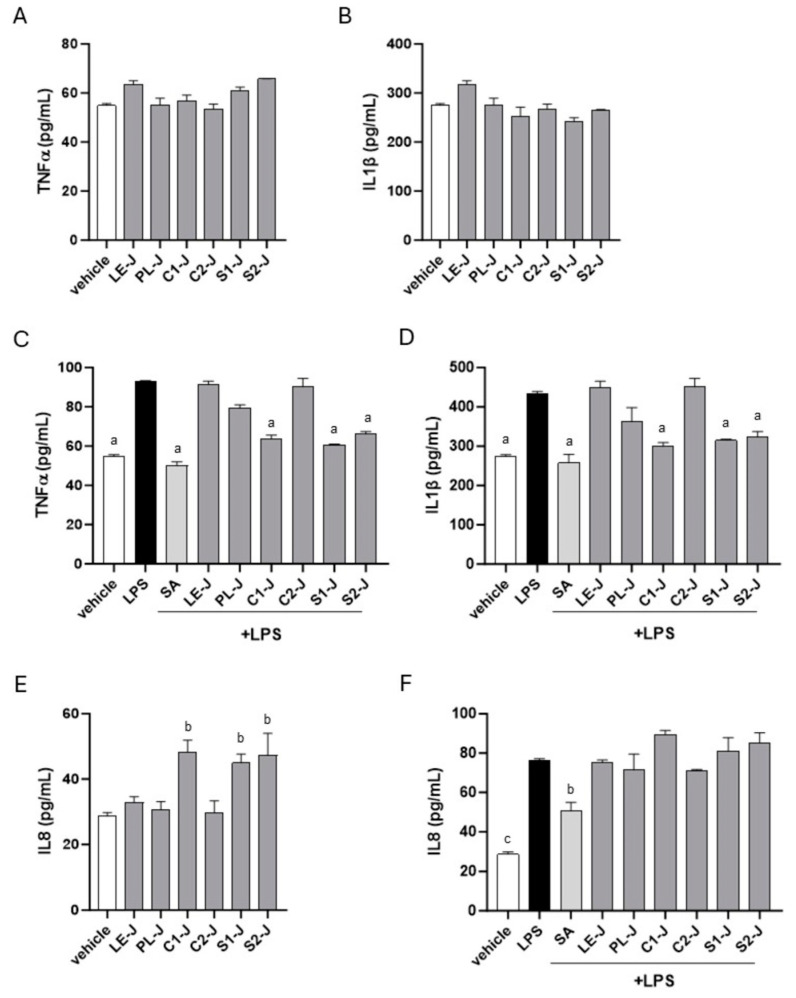
Human primary macrophages were cultured for 24 h with 1% *vol/vol* juices or salicylic acid (SA, 10 nM) in the absence (Panels (**A**,**B**)) or presence (Panels (**C**,**D**)) of LPS 100 ng/mL. The pro-inflammatory cytokines TNF-α (Panels (**A**,**C**)) and IL-1β (Panels (**B**,**D**)) and the chemoattractant factor IL-8 (Panels (**E**,**F**)) were quantified by ELISA in the conditioned media. Data are reported as mean ± SE of three independent experiments, each performed in triplicate. ^a^ denotes *p* < 0.05 vs. LPS, LE-J, and C2-J; ^b^ denotes *p* < 0.05 vs. vehicle-, LE-J-, PL-J-, and C2-J-treated cells; ^c^ denotes *p* < 0.05 vs. all experimental groups.

*S. telephium* juices were tested for anti-inflammatory activity in differentiated primary human macrophages. As reported in Figure 7A,B, juices alone did not activate macrophages toward inflammation. C1-J, S1-J, and S2-J significantly reduced the LPS-induced pro-inflammatory phenotype in macrophages by dampening the production of TNF-α and IL-1β induced by LPS (Figure 7C,D). Consistent with the wound healing results, C1-J, S1-J, and S2-J by themselves increased the production of the chemoattractant factor IL-8 (Figure 7E). Following LPS stimulation, IL8 production increased in all the tested juices compared with vehicle-treated cells, with no statistically relevant differences compared with LPS-treated cells (Figure 7D).

## 3. Discussion

*S*. *telephium* is a well-known plant used for the treatment of inflammation; the compounds primarily responsible for the activity are deemed to be the polysaccharides present at the leaf level [2]. However, several studies have suggested the possible role of the flavonol glycosides in the anti-inflammatory activity [5]; in this paper, we focused our studies on this class of secondary metabolites, investigating their role in both the control of inflammation and healing of the wound [19,20] to eventually support *S. telephium* extracts for the treatment of skin lesions. As far as now, the proposed use of *S*. *telephium* involves applying fresh material, and this approach has several limitations, such as material supply, composition, and applicability in the case of deep wounds. For these reasons, in the present study, we evaluated plant material’s anti-inflammatory and wound-healing activities. The juices of in vivo leaves (LE-J), in vitro grown plantlets (PL-J), undifferentiated calli (C1-J and C2-J), and cell suspensions (S1-J and S2-J) were analyzed, focusing on flavonoidic fraction.

Our results from the qualitative HPLC fingerprint confirmed the results of the literature: only quercetin and kaempferol derivatives were present in *S*. *telephium*. In previous studies, mainly focused on polysaccharide components, only six derivatives of kaempferol and quercetin were identified in the leaf extract, and three glycosylated kaempferol and three glycosylated quercetin forms were found [2]. This study identified 21 derivatives of the two aglycones: 17 kaempferol and 4 quercetin derivatives. Compared with the literature, the extraction method could explain the qualitative richness of our analysis of the juices [2]. Squeezing the material and directly analyzing it could probably avoid the risk of losing compounds due to the extraction and fractionation processes. The qualitative analyses revealed that all the compounds, except compound **5**, were present in LE-J (leaf juice), indicating this juice was the qualitatively richest. Moreover, PL-J showed a qualitatively similar juice but lacked some compounds found in LE-J and also lacked compound **5**. Compound **5** is a quercetin derivative, and we could find it only in C1-J, S1-J, and S2-J (undifferentiated cultures).

LE-J was also the juice with the highest total flavonol content, followed by C1-J and S1-J, PL-J, and S2-J; C2-J showed, instead, the lowest content of total flavonols. The C2-J differed from the other juices obtained from undifferentiated cells and even from its derived suspension (S2-J) both in the low content of flavonoids and the qualitative profile. Our observations could be explained by the absence of hormones in the culture medium, leading to a slowed metabolism, and by considering that calli are a quite heterogeneous population of cells. Indeed, their suspensions, obtained by picking up small clumps of these cell masses, could better develop a homogeneous clone of a fast-growing cell population [21,22]. For these reasons, the total flavonol content and the phytochemical composition of S2-J may overlap with those of other undifferentiated cultures. The cell suspensions are single cells or cell aggregates cultivated under submerged conditions in a liquid medium, where all the cells are in the same nutritious and mixing conditions, leading to a better homogenicity of the products [16]. In our analysis, compound **7**, barely detected in LE-J, and compound **5** were the most representative derivatives of quercetin and indicated an active metabolism of the in vitro undifferentiated cultures, which pursued different metabolic pathways in response to different stress *stimuli* compared with in vivo plants.

Considering the anti-inflammatory potential application of *S*. *telephium* and the Italian ethnobotanical indications for healing scars, burns, ulcers, and wounds, in this study, we aimed to substantiate the use of juices from in vitro cultures, especially from undifferentiated cultures, for wound healing activity. In our study, C1-J, S1-J, and S2-J reported significant anti-inflammatory activity, as demonstrated by the reduction in TNF-α and IL-1β production following stimulation with LPS. The anti-inflammatory activity of juices from *S. telephium* was more or less comparable to the effects of salicylic acid at the in vitro active concentration. Moreover, at the steady state, C1-J, S1-J, and S2-J increased IL8 production in macrophages, inferring a chemoattractant activity able to sustain immune cell recruitment and wound healing [23]. C1-J, S1-J, and S2-J did not affect IL8 secretion under inflammatory conditions (incubation with LPS), at the opposite of salicylic acid (Figure 7F). Nonetheless, C1-J, S1-J, and S2-J promoted wound closure in fibroblasts. Notably, C1-J, S1-J, and S2-J reported the highest content in quercetin derivatives.

It is known that plants containing flavonoids could exert antioxidant and anti-inflammatory activities thanks to the cooperation of this metabolite class and other bioactive compounds that work in combination to heal wounds [24,25,26]. Notably, specific structures or structure modifications can also favor the anti-inflammatory properties of flavonoids, for example, the presence or absence of the unsaturation of the C ring, the carbonyl group on the C-4, the number or the position of the hydroxyls, and the glycosylation. Among the favorable structural arrangements, a catechol group on the B ring confers potent anti-inflammation activity to quercetin [27]. The high content of quercetin derivatives in C1-J, S1-J, and S2-J, together with the presence of a quercetin derivative characteristic of only these juices, support the successfully demonstrated activities, claiming the pivotal role of quercetin in promoting wound healing and anti-inflammatory activity [28]. Both in vivo and in vitro anti-inflammatory activities have also been reported in kaempferol and kaempferol glycosides. Several mechanisms of action mediate the activity, one of which is the inhibition of tumor necrosis factor-alpha (TNF-α) in cells stimulated by LPS [29]. At the same, the kaempferol derivatives detected in the active juices in our study contribute to the whole spectrum of biological activities.

## 4. Material and Methods

### 4.1. Plant Material

Leaves of *Sedum telephium* were harvested from cultivated species at full flowering. The taxonomic identification of the plant was carried out by Dr. G. Cassina. The voucher specimen (DSF-PD-ST-1-18) was deposited in the Plant Biotechnology laboratory of the Department of Pharmaceutical and Pharmacological Sciences (University of Padova, Padova, Italy).

### 4.2. In Vitro Cultures

Callus cultures were obtained from leaf explants cultured on Schenk and Hildebrandt basal medium [30] containing 30 g/L of sucrose and 2 mg/L of 2,4-dichlorophenoxyacetic acid combined with 2 mg/L of kinetin (S10 medium, hereafter). The medium was solidified with agar (10 g/L); the pH was adjusted to 5.7. Cultures were established in 9 cm Petri dishes, using 8 explants per dish. The cultures were maintained in a tissue culture chamber at a temperature of 25 °C under cool white fluorescent lights (36 mol m^−2^ s^−1^) at 16 h photoperiod and subcultured on fresh media every 5 weeks. After 10 subcultures, calli were partially subcultured in the same medium without hormones (SH medium, hereafter) and maintained in the same culture conditions.

Calli cultured on S10 and SH media were used to establish shake-flask suspension cultures using liquid media with the same composition as above. Calli (5 g fresh weight) were transferred into liquid medium (100 mL) in 500 mL Erlenmeyer flasks, maintained at 110 rpm in a rotary shaker, and subcultured every 14 days.

Stabilized calli (over 20 subcultures) and cells from suspension cultures (over 5 subcultures) were collected on the 20^th^ and 8^th^ days of the growth cycle, respectively.

Plantlets cultured on hormone-free solid medium Murashige and Skoog [31] (MSHF, hereafter) obtained by indirect organogenesis from calli were collected when they reached around 4 cm high.

### 4.3. Material Extraction

**Figure 8:**
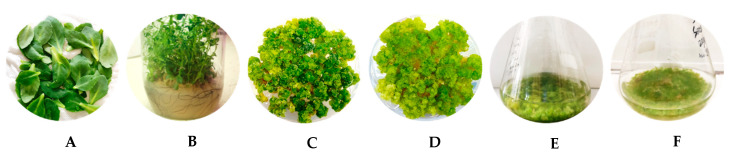
Plant materials used for sample preparations. Leaves from in vivo plants (**A**), plantlets grown in vitro (**B**), calli in S10 medium (**C**), calli in SH medium (**D**), cells of suspension cultures in S10 (**E**), and cells of suspension cultures in SH (**F**).

Leaves from in vivo plants, plantlets cultured on MSHF, calli, and cells from suspension cultures cultured in S10 and SH media were used (Figure 8).

The collected materials were first frozen −18 °C, then, once thawed, were squeezed (adding quartz powder) with a micropestle. The samples were transferred in the ultrasound bath for 40 min. After centrifugation (13,200 rpm), the supernatant of each sample (juice) was taken and analyzed. The juice samples were named as follows: LE-J (leaves from in vivo plants), PL-J (plantlets cultured on MSHF), C1-J (calli cultured in S10), C2-J (calli cultured in SH), S1-J (cell suspension in S10), and S2-J (cell suspension in SH).

### 4.4. Juice Acidic Hydrolysis

The juice hydrolyses were performed according to [32]. Briefly, the juices were added with HCl 6 M, placed in a water bath, and heated in a discontinuous way (on/off 15 s) in a microwave oven (800 w) for 30 min. After cooling, a liquid/liquid extraction with diethyl ether was performed twice; the ether extracts were evaporated under reduced pressure to dryness, and the residues were dissolved in methanol.

### 4.5. Chemicals

HPLC-grade methanol and acetonitrile, analytical-grade acetic acid, and ethyl ether were purchased from Sigma-Aldrich (Milan, Italy). Ultrapure water was used, and it was obtained using a Milli-Q^®^ dispenser (Merck, Darmstadt, Germany). Kaempferol and quercetin reference standards were purchased from Sigma-Aldrich (Milan, Italy).

### 4.6. Chemical Analysis

HPLC-DAD analyses were performed using an Agilent 1100 HPLC Series System (Agilent, Santa Clara, CA, USA) equipped with a degasser, quaternary gradient pump, column thermostat, and UV-Vis detector. A Gemini 5 µm C6-Phenyl column (250 × 4.6 mm) from Phenomenex (Torrance, CA, USA) was employed at 40 °C. The mobile phase consisted of 0.15% acetic acid in water (A) and acetonitrile (B), with the following gradient elution program: 97% A at 0–6 min, 75% A at 15 min, 75% A at 20 min, 20% A at 30 min, and 97% A at 40 min. The flow rate was 1 mL/min, with an injection volume of 10 µL; chromatograms were acquired at 265 and 365 nm; and UV–Vis spectra were recorded in the 190–700 nm range [33].

For the quantification of flavonols, the chromatograms acquired at the wavelength of 365 nm were used. The content was expressed as quercetin and kaempferol derivatives using authentic commercial standards. Quercetin and kaempferol standard solutions (1 mg/mL) were prepared in methanol, and the calibration curves were obtained in a concentration range of 2–50 μg/mL, with six concentration levels. Peak areas were plotted against corresponding concentrations (kaempferol: R^2^ = 0.9997; quercetin: R^2^ = 0.9992). The analysis was performed in triplicate, and the results were expressed as mean ± standard deviation (SD).

### 4.7. Cell Culture Conditions

Primary human macrophages were prepared from buffy coats obtained and stored at the blood bank of the Padova University Hospital. The study protocol was reviewed and approved by the local Hospital’s Ethics Committee (registration number CE: 091/2016). The buffy coat was processed as previously described [34]. Briefly, buffy coat was mixed 1:1 (*vol*/*vol*) with sterile RPMI 1640 Medium (Life Technologies, Monza, Italy) and layered over Ficoll-Paque PLUS (Merck, Milan, Italy). Samples were centrifuged (1200 rpm, 30 min), and peripheral blood mononuclear cells (PBMCs) were collected. Cells were washed (1600 rpm, 10 min) and suspended in RPMI 1640 supplemented with 1% (*vol*/*vol*) penicillin and streptomycin, 2 mM L-glutamine, and 10% heat-inactivated fetal bovine serum (FBS; Life Technologies). PBMCs were counted and cultured (3 × 10^6^ cells/mL) in tissue culture plates (Corning; Merck, Milan, Italy) for 3 h at 37 °C and 5% CO_2_ in a humidified incubator. Floating cells were then removed, whereas attached cells were washed and differentiated in mature macrophages by incubating them for ten days in culture media supplemented with recombinant human granulocyte-macrophage colony-stimulating factor (rhGM-CSF, 2 ng/mL; ImmunoTools; Friesoythe, Germany). The culture media were renewed every three days.

The human foreskin fibroblasts 1 (HFF1) cell line was purchased from the American Type Culture Collection (ATCC, LGC Standards S.r.l., Sesto San Giovanni, Italy) and routinely cultured in DMEM supplemented with 1% (*vol*/*vol*) penicillin and streptomycin, 2 mM L-glutamine, and 10% (*vol*/*vol*) heat-inactivated FBS (all purchased from Life Technologies). Cells were grown at 37 °C and 5% CO_2_ and detached using Trypsin-EDTA (Life Technologies). Cells were used at passages 5–7 and seeded at 1 × 10^5^ cells/mL for the experiments described.

### 4.8. Cell Viability Assay

Differentiated primary macrophages were cultured in 96-well tissue culture plates (100 μL culture media) and incubated for 24 h with *S. telephium* juices at the final concentrations ranging from 0.2 to 25% *vol*/*vol*. Control cells were incubated with the diluent media (cell culture media). At the end of incubation, the cytotoxicity was evaluated using the MTT (3-(4,5-Dimethylthiazol-2-yl)-2,5-Diphenyltetrazolium Bromide) assay as previously reported [35]. Briefly, cell cultures were washed and incubated at 37 °C for 4 h with MTT solution (5 mg/mL, Merck). Formazan crystals were then solubilized in 100 µL of SDS 10% *w*/*vol*, HCl 0.01 N. The absorbance was recorded 16 h later at 590 nm using a microplate reader (MultiPlateReader VictorX2, Perkin Elmer, Milan, Italy). The viability of treated cells versus vehicle-treated cells was calculated as follows: % cell viability = (OD_590nm_ treated cells) × 100/(OD_590nm_ vehicle).

### 4.9. Wound Healing Assay

HFF1 cells were seeded on 6-well tissue culture plates in 2 mL complete media and cultured for 24 h. Then, a scratch was made using a 1000 μL pipette tip, as previously described [3,36]. The cell cultures were washed and incubated in culture media supplemented with *S. telephium* juices at 1% *vol*/*vol* or 10 ng/mL bFGF (Gibco, New York, NY, USA). The cultures were stopped 24 h later. For each sample, three different areas along the scratches were analyzed by optical microscopy. The distance between cells at the edges of the scratch was measured using the software ImageJ and expressed as the percentage of closure of the area as compared with cells incubated with the vehicle alone.

### 4.10. Enzyme-Linked Immunosorbent Assay

Differentiated primary human macrophages cultured in 96-well tissue culture plates were incubated for 24 h with *S. telephium* juices at the final concentrations of 1% *vol/vol* with or without 100 ng/mL lipopolysaccharide (LPS, from *Salmonella enterica* serotype Typhimurium; Merck). As a positive control, macrophages were treated with LPS 100 ng/mL and salicylic acid 10 nM (a concentration previously identified as effective).

At the end of incubation, Tumor Necrosis Factor (TNF)-α, Interleukin (IL)-1β, and IL-8 were quantified in the conditioned media using commercially available enzyme-linked immunosorbent assay kits (ELISA, Affymetrix eBioscience; Prodotti Gianni, Milan, Italy) [37]. Optical densities were measured at 450 nm using a microplate reader (MultiPlateReader VictorX2, Perkin Elmer). The sensitivity of the assays was in the range of 10–15 pg/mL. Experiments were performed in triplicate.

### 4.11. Statistical Analyses

Results are reported as mean ± standard deviation (SD) for chemical investigations and as mean ± the standard error of the mean (SE) for biological investigations. Statistical analysis was performed using the one-way ANOVA test followed by the Newman–Keuls post hoc test, using GraphPad Prism 3.03 (San Diego, CA, USA). *p* values < 0.05 were considered statistically significant.

## 5. Conclusions

Among plants well known for their anti-inflammation activity, *Sedum telephium* is an ethnobotanical source that is useful in treating skin injury. The present study aimed to evaluate the use of in vitro cell cultures of *S*. *telephium*, as a biotechnological tool to overcome the problem related to the traditional preparation protocols. The possibility of obtaining high-yielding cell lines with a favorable biosynthetic profile could be an intriguing challenge, considering the peculiar characteristics of in vitro material, such as the absence of pollutants, contaminants, and microorganisms. Moreover, the fact that in vitro cell cultures have no known negative impact on ecosystems and the environment by avoiding competition for arable land makes them worthy of study. The results of this study highlight the great potential of *S*. *telephium* in vitro cell cultures, especially undifferentiated in vitro cultures, as sources of plant material endowed with anti-inflammatory and wound healing properties.

## Figures and Tables

**Table 1:** Kaempferol derivatives (black) and quercetin derivatives (red) in LE-J, PL-J, C1-J, C2-J, S1-J, and S2-J. LE-J, juice from in vivo leaves; PL-J, in vitro plantlets; C1-J, calli cultured in S10; C2-J, calli cultured in SH; S1-J, cell suspension in S10; S2-J, cell suspension in SH.

Compound (RT)	1 (15.6)	2 (15.7)	3 (16.0)	4 (16.8)	5 (17.1)	6 (17.3)	7 (17.7)	8 (17.8)	9 (18.0)	10 (18.1)	11 (18.4)	12 (18.6)	13 (19.0)	14 (19.2)	15 (19.5)	16 (20.3)	17 (22.7)	18 (24.9)	19 (25.6)	20 (26.1)	21 (27.0)
LE-J	X	X	X	X	-	X	X	X	X	X	X	X	X	X	X	X	X	X	X	X	X
PL-J	X	X	X	X	-	X	-	X	X	X	X	X	X	X	X	-	X	-	-	-	X
C1-J	X	-	X	X	X	X	X	-	-	-	-	-	-	-	-	-	-	-	-	X	X
C2-J	X	X	X	X	-	X	-	-	-	-	-	-	-	-	-	-	-	-	-	X	-
S1-J	-	-	X	X	X	X	X	-	-	-	-	-	-	-	-	-	-	-	-	X	-
S2-J	X	-	X	-	X	X	X	-	-	-	-	-	-	-	-	-	-	-	-	X	-

## Data Availability

The data presented in this study are available on request from the authors.
